# Cranial and Cervical Muscular Weakness in Mitochondrial Myopathy Is Associated With Resolution of Migraine Headaches: Further Evidence That Muscular Compression of Cranial and Peripheral Nerves Is a Cause of Headache in a Subset of Patients With Migraine

**Published:** 2015-06-08

**Authors:** Edward P. Miranda

**Affiliations:** ^a^Center for Complex Reconstruction, San Francisco, Calif; ^b^Department of Plastic Surgery, California Pacific Medical Center, San Francisco, Calif

**Keywords:** migraine, peripheral nerve, cranial nerve, trigeminal, mitochondrial myopathy

## Abstract

**Objective:** A significant subset of patients with migraine headaches has pain relief after neuroplasty/muscular decompression of select cranial and cervical nerves. In the majority of cases, compression occurs secondary to compression of the nerves by adjacent muscles. Previous studies have shown that both surgical decompression and chemical denervation (eg, botulinum toxin) provide relief of migraine headaches; however, controversy remains. If some migraine headaches are caused by muscular compression, then paresis of the compressing muscles by underlying myopathic/metabolic disease should result in migraine relief in some patients. **Methods:** We report a case of mitochondrial myopathy causing weakness primarily of the muscles of facial expression and the neck in the context of chronic migraine headaches (>20-year history). Muscle biopsy was obtained to confirm the myopathic diagnosis. **Results:** There was complete resolution of the patient's migraine headaches that occurred simultaneously with the onset of symptomatic paresis of the muscles of facial expression and the neck. The relief has persisted for more than 10 months. Neurologic evaluation and muscle biopsy confirmed a diagnosis of mitochondrial myopathy. **Conclusions:** Pathologic paresis/paralysis of facial and/or cervical muscles can result in persistent resolution of migraine headache pain, giving further evidence to the concept that peripheral and/or cranial nerve compression causes migraine headache pain in a subset of patients with a diagnosis of migraine.

There is a growing body of evidence that cranial and peripheral nerve compression causes headache pain in a subset of patients with a diagnosis of migraine.^1^ Conversely, decompression/neuroplasty of compressed nerves can provide objective headache relief in 80% to 90% of select patients.^2^ The use of botulinum toxin has been reported to reduce the frequency and intensity of migraine headaches when administered to compressing muscles.^3^ If therapeutic paresis/paralysis of select muscles can cause migraine relief, the development of underlying metabolic disease, myopathy, and/or neuropathy causing weakness in similar muscle groups should also give migraine relief in at least some patients with migraine. We report a case of chronic migraine headaches that resolved contemporaneously with the development of symptomatic facial and cervical weakness due to mitochondrial myopathy.

## METHODS/CASE REPORT

A 46-year-old woman with a history of migraine headaches, closed head injury, and temporomandibular joint dysfunction after jaw dislocation presented with progressive weakness, particularly in the face, head, neck, and proximal upper extremities. The weakness was first noted 10 months earlier. The weakness was initially noted in the right upper eyelid (ptosis) and the right face. The facial weakness then progressed to bilateral facial weakness. There was no history of botulinum toxin treatment.

## RESULTS

Physical examination revealed cranial motor weakness, including brow ptosis, eyelid ptosis, and the absence of corrugator or procerus function bilaterally. Jaw strength was normal.

Further inquiry into her neurologic history revealed that her migraine headaches primarily initiated with pain in the supraorbital areas bilaterally with tenderness over the supraorbital notches. The patient reported frequent migraine headaches that began in her third decade of life. She reported at least 2 disabling migraines per month. The migraines worsened in intensity in her fourth and fifth decades. Contemporaneously, with the development of symptomatic facial and cervical weakness (10 months prior to evaluation), the patient's migraines ceased. This resolution has persisted over the following 10 months.

Initially, myasthenia gravis was considered, but testing was negative. A deltoid muscle biopsy was performed. Biopsy results were consistent with mitochondrial myopathy.

## DISCUSSION

Compression of cranial and cervical nerves occurring in the context of migraine headaches and the relief of migraine headaches by chemical or surgical decompression of these nerves have been a subject of significant study over the past few years. Decompression/neuroplasty of compressed nerves can provide objective headache relief in 80% to 90% of select patients and has been demonstrated in prospective, placebo controlled trials of the supraorbital and supratrochlear nerves (trigeminal V1 subdivisions), the zygomaticotemporal nerve (trigeminal V2 subdivision), and the greater occipital nerve (C2 subdivision).^2^ Further data exist supporting the compressive mechanism in other nerves, including the lesser and third occipital nerves, the auriculotemporal nerve, and others.

In the overwhelming majority of cases with a compressive neuropathy mechanism, the compression is caused by muscle (eg, corrugators compressing the supraorbital nerve), but to a lesser extent there are other causes including bony and cartilaginous impingement (eg, the presence of a supraorbital foramen rather than a notch and nasal septum-turbinate contact, respectively).^4^ Consequently and somewhat predictively, pharmacologic paralysis of targeted muscles will relieve migraine headache pain if applied selectively or in a broad “shotgun-approach” protocol.^3^

Nevertheless and despite these data, controversy exists over the role and adequacy of these trials and reports.^5^ Given the superficial and observable nature of some of the treated muscles (eg, the corrugators), some investigators argue that a true blinded or placebo study is not possible. To some extent (and at some sites), these objections are valid, as the nature of either surgical correction of the corrugators or botulinum toxin–mediated paresis is easily observable. However, at other major sites (greater occipital nerve, zygomaticotemporal branch of the trigeminal nerve, etc), it is less certain that the lay observer could distinguish placebo versus treatment.

This case report approaches this controversy from a different perspective: namely, a patient with established migraine headaches that resolved after development of craniofacial and cervical muscle weakness due to muscle biopsy–proven mitochondrial myopathy.^6^ As there were no intentions to treat the migraine disease, placebo-like effects should not come into play in this example. In the described patient, her migraines initiated regularly at the supraorbital notches, suggesting compressive neuritis of the supraorbital nerves. The resolution of the migraines at the same time as the development of brow and corrugator weakness lends significant support to the nerve compression theory.

This case is the first case to our knowledge that reports migraine resolution in the context of the development of myopathic disease. It generates questions for further research such as what is the frequency of migraine resolution/improvement in other cases of nontraumatic pathologic facial/cervical weakness.
